# Bisphenol S Impaired In Vitro Ovine Early Developmental Oocyte Competence

**DOI:** 10.3390/ijms21041238

**Published:** 2020-02-12

**Authors:** Alice Desmarchais, Ophélie Téteau, Pascal Papillier, Manon Jaubert, Xavier Druart, Aurélien Binet, Virginie Maillard, Sebastien Elis

**Affiliations:** 1 UMR PRC, CNRS, IFCE, INRAE, Université de Tours, 37380 Nouzilly, France; alice.desmarchais@inra.fr (A.D.); ophelie.teteau@inra.fr (O.T.); pascal.papillier@inra.fr (P.P.); jaubert.manon@gmail.com (M.J.); xavier.druart@inra.fr (X.D.); aurelien.binet@univ-tours.fr (A.B.); virginie.maillard@inra.fr (V.M.); 2 CHRU de Tours, 37000 Tours, France

**Keywords:** endocrine disruptors, Bisphenol S, oocyte competence, ewe

## Abstract

Introduction: Bisphenol A (BPA) is a widespread compound in the plastic industry that is especially used to produce baby bottles, food packaging and metal cans. BPA, an endocrine disruptor, leads to alterations in reproductive function and therefore has been banned from the food industry. Unregulated BPA analogues, particularly Bisphenol S (BPS), have emerged and are now used in the plastic industry. Thus, this study aimed to examine the acute effects of low and environmental doses of BPS on ewe oocyte quality and developmental competence, and its mechanism of action, during in vitro maturation. Methods: Ewe cumulus-oocyte complexes underwent in vitro maturation in the presence or absence of BPS (1 nM, 10 nM, 100 nM, 1 µM or 10 µM). Oocytes were then subjected to in vitro fertilisation and development. Results: 1 µM BPS induced a 12.7% decrease in the cleavage rate (*p* = 0.004) and a 42.6% decrease in the blastocyst rate (*p* = 0.017) compared to control. The blastocyst rate reduction was also observed with 10 nM BPS. Furthermore, 10 µM BPS reduced the oocyte maturation rate, and 1 µM BPS decreased cumulus cell progesterone secretion. *PR* and *AMH* gene expression were reduced in cumulus cells. BPS induced a 5-fold increase in MAPK 3/1 activation (*p* = 0.04). Conclusions: BPS impaired ewe oocyte developmental competence. The data suggest that BPS might not be a safe BPA analogue. Further studies are required to elucidate its detailed mechanism of action.

## 1. Introduction

Bisphenol A (BPA) is a widespread compound that is used in the plastic industry to produce medical devices, baby bottles, food packaging, metal cans and cash receipt coatings [1]. Diet is therefore the main source of human BPA contamination [2,3]. This widespread use led to the detection of BPA in 95% of patient urine samples in the United States at concentrations ≥ 0.1 ng/mL (0.44 nM) [4], with an average urine and blood concentration of 1–3 ng/mL (4–13 nM) [1]. BPA has also been detected in amniotic fluid (1–9 ng/mL), follicular fluid (2.4 ± 0.8 ng/mL), neonatal, placenta and foetal blood (2.2 ± 1.8 ng/mL), human breast milk (approximately 1 ng/mL) and adipose tissue (1.8–12 ng/g) [5,6,7]. These data indicate that critical exposure windows occur during pregnancy or infancy.

Both in vitro and in vivo studies demonstrated that BPA has deleterious effect on health [8,9,10]. Indeed, nanomolar doses of BPA are associated with obesity, cardiovascular diseases [10,11], type 2 diabetes [11,12] and alterations in reproductive function [13]. BPA is classified as a weak estrogen [14], with a 10,000-fold lower affinity for estrogen receptor (ER) α and ERβ receptors than 17β oestradiol (E2) [15,16]. Moreover, among women who are undergoing in vitro fertilisation (IVF), the highest urinary BPA concentrations (nanomolar range) are associated with decreased number and quality of oocytes and decreased E2 levels [17,18,19]. BPA also reportedly disrupts steroid production in rat and porcine granulosa cells [20,21,22] and cumulus–oocyte complex (COC) [23]. Both folliculogenesis and oocyte maturation (meiotic abnormalities) are affected by BPA [24,25].

After BPA was banned from the food industry [26,27], unregulated BPA analogues, particularly Bisphenol S (BPS), have emerged and are used in the plastic industry. BPS is therefore now detected in urine at the same concentration range as BPA (0.02–21 ng/mL or 0.09–91 nM) [28]. Due to their analogous structures, BPS and BPA are suggested to exhibit similar properties and adverse health effects. Some studies in fish and rodent species reported that BPS affects germinal cells and endocrine function similarly to BPA [29,30,31]. In zebrafish, chronic exposure of BPS for 75 days leads to increased plasma oestradiol levels and decreased egg production and sperm count [32]. In female rats, neonatal exposure to BPA or BPS during 10 days delays puberty onset and oestrous cyclicity [33]. In mice, 4 week BPS treatment decreased ovary weight and the number of primary, preantral and antral follicles [34]. Such a prolonged exposure increases spindle malformation and chromosome misalignment in mice oocytes reduces cleavage rate [34] and reduces blastocyst rate [35]. Some studies showed BPS treatment during in vitro maturation alters metaphase II achievement in porcine oocyte [36] and modifies spindle morphology and chromosome alignment in bovine matured oocytes [37]. In a sensitive in vitro foetal model, human gonadal cells were 10- to 100-fold more sensitive to BPA, BPS and Bisphenol F (BPF) than rodent cells [1]. It is thus necessary to assess the BPS effects in a mono-ovulating mammalian species like the ewe, as it is a relevant model for bisphenols toxicological studies [38,39,40] and for human reproductive physiology [41,42]. BPS is now detected in women physiological fluids and, BPS exposure is even rising [43]. However, toxicological studies usually examine endocrine disruptors such as BPA at high doses [44] while outcomes are described at a much lower dose [45].

Therefore, this study aimed to study the acute *in vitro* effects of low and environmental doses of BPS on ewe oocyte quality and developmental competence, and its mechanism of action, during *in vitro* maturation (IVM). *In vitro* fertilisation (IVF) was performed after 24-h oocyte maturation in the presence of BPS. First, the effect of five BPS doses was assessed on cleavage and blastocyst developmental rates and embryo cell numbers, including both environmental doses (1 nM, 10 nM and 100 nM) and supraenvironmental doses (1 µM and 10 µM) because the duration of *in vitro* exposure is short. We purposefully chose to study BPS effects in an oestradiol-containing medium, even knowing that oestradiol could modulate BPS effects. Indeed, the oocyte is physiologically surrounded by follicular fluid, which contained high concentrations of oestradiol, especially during late maturation stage. Therefore, studying BPS effects on the oocyte, regardless of oestradiol presence, appeared irrelevant to physiology and would not provide any information on its potential *in vivo* effects. Then, we investigated whether the effects observed on developmental rates could be explained by other parameters. The BPS concentrations affecting developmental rates were then assessed on COC viability, maturation and the oocyte MAPK3/1 signalling pathway. Taking into account that cumulus cells (CC), enclosing the oocyte, are important in terms of both bidirectional molecule exchange and oocyte protection [46,47], CC progesterone secretion and gene expression were also investigated.

## 2. Results

### 2.1. Low-Dose BPS Treatment During IVM and Effects on in vitro Embryo Development (IVD)

**Figure 1:**
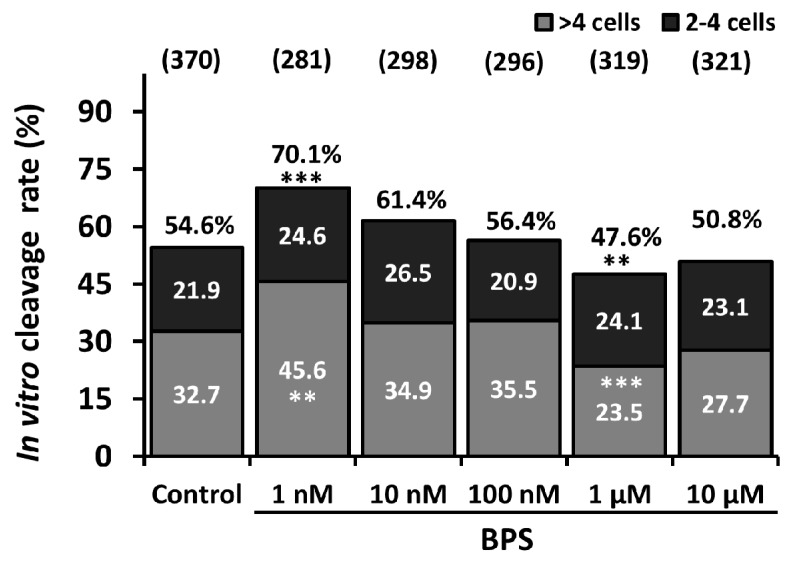
Effect of Bisphenol S (BPS) during *in vitro* maturation (IVM) on the cleavage rate. Oocytes underwent IVM in the presence or absence of BPS (1 nM, 10 nM, 100 nM, 1 µM or 10 µM). Oocytes were then subjected to in vitro fertilisation (IVF), and the cleavage rate was assessed 2 days after IVF. Embryos with more than four cells were distinguished among mature and fertilised cumulus oocyte complex (COCs). The percentage of cleaved embryos was normalised to the total number of oocytes that underwent IVF. The results from eight independent experiments are expressed as the raw percentage of embryos at each developmental stage. ** indicates a significant difference for a given stage compared with the control (*p* ≤ 0.01). *** indicates a significant difference for a given stage compared with the control (*p* ≤ 0.001). Total numbers of mature and fertilised COCs per group are indicated between parentheses.

After COC IVM in the presence or absence of BPS (1nM, 10 nM, 100 nM, 1 µM or 10 µM) and IVF, embryo rates were analysed after 2 or 7 days of development (Supplementary Figure S1). The embryo cleavage rate was determined among matured and fertilised COC at day 2 of IVD. As shown in Figure 1, the raw cleavage rate significantly increased by 28.4% with 1 nM BPS (*p* = 0.0003) and decreased by 12.7% with 1 µM BPS (*p* = 0.004) compared to control (54.6%). Ten micromolar BPS also tended to decrease the cleavage rate by 7% (*p* = 0.09) compared to the 0 h control.

Cleaved embryos were classified as either 2–4 cell embryos or > 4-cell embryos. There were no differences in the 2–4-cell embryo rate for any conditions compared to the control. On the contrary, 1 µM BPS significantly decreased the > 4-cell embryo rate by 28% compared to control (32.7%) and 10 µM BPS tended to decrease the > 4 cell embryo rate by 15.2% (*p* = 0.06).

**Figure 2:**
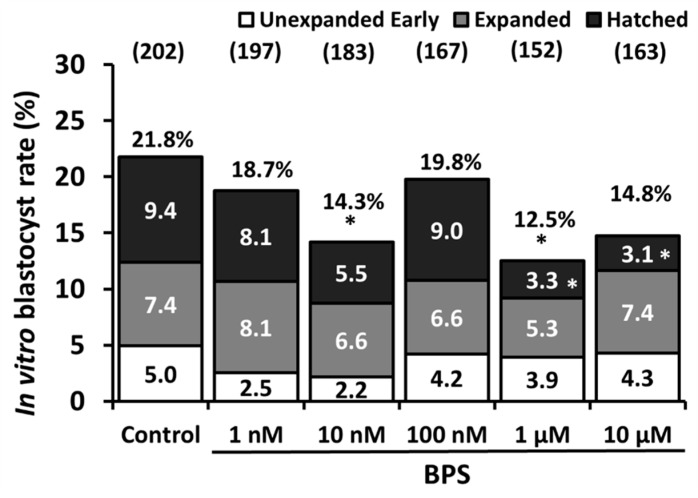
Effect of Bisphenol S (BPS) during in vitro maturation (IVM) on the blastocyst rate. Oocytes underwent IVM in the presence or absence of BPS (1 nM, 10 nM, 100 nM, 1 µM or 10 µM). Oocytes were then subjected to *in vitro* fertilisation (IVF), and the blastocyst rates were assessed 7 days after IVF. Blastocysts were classified as unexpanded early, expanded or hatched. Raw percentages were derived from the number of blastocysts normalised to the number of cleaved embryos at day 2. The results from eight independent experiments are expressed as the raw percentage of embryos at each developmental stage. * indicates a significant difference for a given stage compared with the control (*p* ≤ 0.05). Numbers of cleaved embryos at day 2 are indicated between parentheses.

At day 7 of IVD, the blastocyst rate was evaluated among cleaved embryos (Figure 2). Ten nanomolar and 1 µM BPS significantly reduced the blastocyst rate by 34.4% (*p* = 0.046) and 42.7% (*p* = 0.017), respectively, compared to control (21.8%). Blastocysts were classified as unexpanded early, expanded or hatched (Supplementary Figure S1). One and ten micromolar BPS significantly reduced the hatched blastocyst rates by 64.9% (*p* = 0.0315) and 67% (*p* = 0.021), respectively, compared to the control rate (5.0%).

Because BPS 1 nM and 1 µM altered cleavage rate, these concentrations, as well as the highest one, BPS 10 µM, were chosen to investigate oocyte maturation. Concerning all other experiments, because BPS 10 nM, 1 µM and 10 µM altered oocyte developmental competence, these three concentrations were chosen to investigate whether effects on viability, cumulus cell gene expression, and oocyte MAPK3/1 signalling pathway could explain the effects on oocyte developmental competence.

### 2.2. BPS Effect on COC Viability

**Figure 3:**
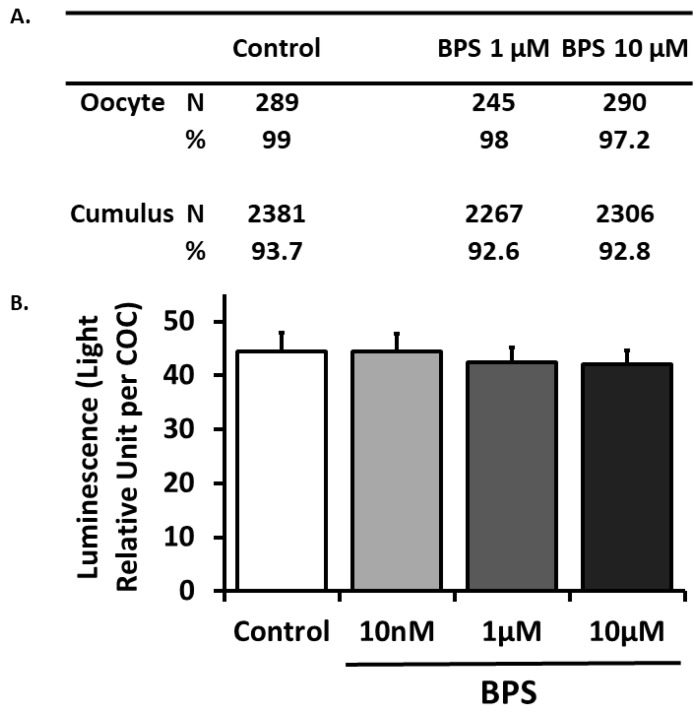
Effect of Bisphenol S (BPS) during in vitro maturation (IVM) on cumulus–oocyte complex (COC) viability. COCs underwent IVM in presence or absence of BPS (10 nM, 1 µM or 10 µM). (**A**) Cumulus cell (CC) and oocyte viability were assessed using the Live/Dead Viability/Cytotoxicity Kit for mammalian cells after removal of surrounding CCs. Results of three independent experiments with 50–60 COCs per condition are expressed as percentage of living cells in each condition. (**B**) COC viability was assessed on spent 24 h IVM media using the Bioluminescence Cytotoxicity Assay Kit. Data are expressed as the mean ± SEM of 11 independent cultures in light relative units per COC.

BPS effects on cell viability were investigated using cell live/dead staining (Figure 3A) and adenylate kinase assay in spent media (Figure 3B). The living oocyte rate evaluated by live/dead staining was 99% for the control condition and was not affected by 1 or 10 µM BPS treatment (*p* = 0.48 and *p* = 0.136, respectively). The living cumulus cell rate was 93.7% for the control condition and was not affected by 1 or 10 µM BPS (*p* = 0.577 and *p* = 0.812, respectively). COC viability was also assessed by measuring adenylate kinase activity in spent *IVM* culture media from the corresponding COC (Figure 3B). There were no significant differences in the levels among control and BPS conditions (10 nM, 1 µM and 10 µM).

### 2.3. BPS Effect on Oocyte Maturation

The oocyte nuclear maturation stage was evaluated after 24 h IVM in the presence or absence of BPS (1nM, 1µM or 10 µM). A mature state (mix of telophase I and metaphase II) was reached by 88.2% of oocytes in the control condition (Table 1). There were no differences in maturation stage for 1 nM and 1 µM BPS. However, 10 µM BPS significantly decreased metaphase II maturation by 15% (76.7%, *p* = 0.008) compared to the control.

### 2.4. 24-h IVM BPS Treatment Effect on Day 6 Embryo Cell Number

There was already a huge variability in embryo stages at day 7 IVD, from unexpanded blastocysts to hatched blastocysts, and thus the number of cells (Table 2) was next determined at day 6 post-IVF in morula originated from 10 nM and 1 µM BPS-treated oocytes during 24 h IVM. There were no differences in morula cell numbers among the control condition (28.3 ± 2.9 cells), 10 nM BPS (33.1 ± 3.4 cells) and 1 µM BPS (22.2 ± 2.3 cells).

### 2.5. BPS Effect on COC Progesterone Secretion During 24-h IVM

**Figure 4:**
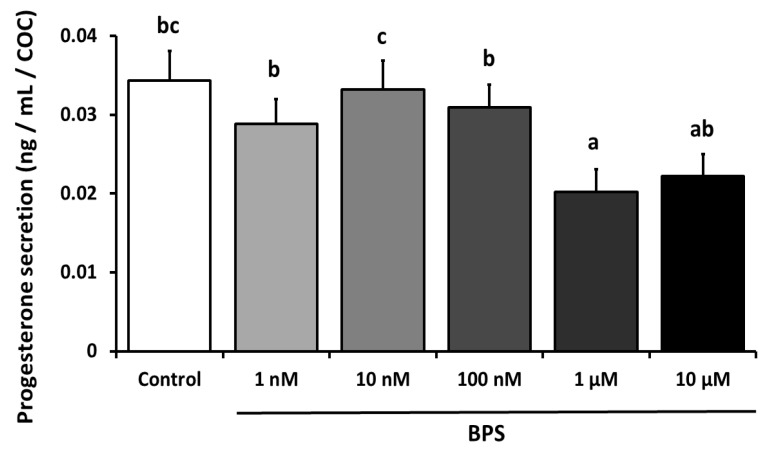
Effect of Bisphenol S (BPS) on progesterone secretion by cumulus oocyte complex (COCs) after 24 h *in vitro* maturation (IVM). COCs underwent IVM for 24 h in the presence or absence of BPS (1 nM, 10 nM, 100 nM, 1 µM or 10 µM). The progesterone concentration was measured in IVM media, and its value was normalised by the number of COCs in each well. Data are expressed as ng progesterone secreted per COC. The results of six independent experiments are presented as mean ± SEM. Bars with different superscripts indicate significant difference (*p* ≤ 0.05).

Progesterone was measured in 24 h IVM spent culture media (Figure 4). There was a significant 41% decrease (*p* < 0.0001) in progesterone secretion with 1 µM BPS (0.02 ng/mL/COC) compared to the control condition (0.034 ng/mL/COC). There were no differences with any other BPS concentrations.

### 2.6. BPS Effect on Cumulus Cell mRNA Expression and on Oocyte MAPK3/1 Phosphorylation After 6 h IVM

Based on the results obtained on blastocyst rates (Figure 2), expression of 11 genes (Table 3), including steroid and hormonal receptors (*ESR1*, *ESR2*, *GPER*, *PR*, *AR*, *FSHR* and *AMHR2*), ovarian reserve marker (*AMH*), bone morphogenetic protein receptors (*BMPR1A* and *BMPR1B*) and a transcription factor (*PPARG*), was measured in CC using qPCR after 6 h IVM in presence or absence of BPS (10 nM, 1 µM).

**Figure 5:**
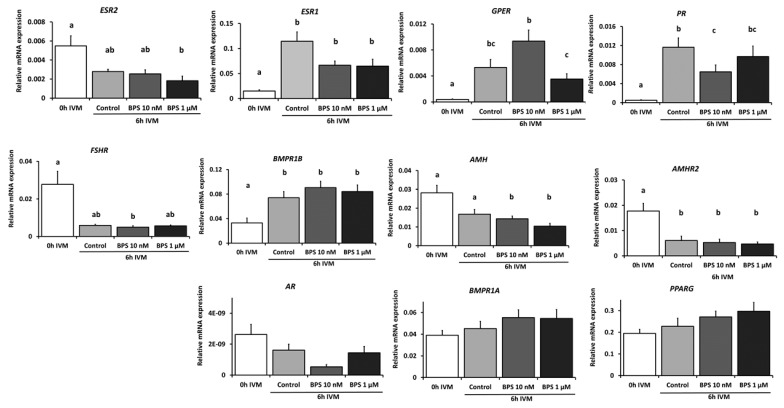
Gene expression in cumulus cells before and after 6 h in vitro maturation (IVM) with BPS treatment. Cumulus oocyte complex (COC) underwent IVM for 6 h in the presence or absence of BPS (1 nM, 10 nM, 100 nM, 1 µM or 10 µM). Total RNA was extracted from cumulus cells and reverse transcribed, and qPCR was performed. Relative gene expression of *ESR1, ESR2, GPER, PR, AR, AMH, AMHR2, BMPR1A, BMPR1B, FSHR*, and *PPARG* was measured in cumulus cells. The geometric mean of two housekeeping genes (*RPL19* and *ACTB*) was used to normalise gene expression. Results of seven to ten independent samples per condition are presented as mean ± SEM. Bars with different superscripts indicate significant difference (*p* ≤ 0.05).

As shown in Figure 5, *ESR1*, *GPER*, *PR* and *BMPR1B* expression significantly increased after 6-h IVM (*p* ≤ 0.0001, *p* ≤ 0.001, *p* ≤ 0.001 and *p* ≤ 0.01, respectively). On the contrary, *AMHR2* expression was significantly decreased (*p* ≤ 0.001) after 6 h IVM. The expression of the six remaining genes (*ESR2*, *AR*, *AMH*, *BMPR1A*, *FSHR* and *PPARG*) did not vary after 6 h IVM. Regarding BPS effects, 10 nM BPS significantly decreased relative *PR* mRNA expression by 45.5% (*p* ≤ 0.001) compared to the 6 h IVM control. Moreover, 1 µM BPS significantly decreased relative *ESR2* mRNA expression by 66.7% (*p* ≤ 0.001) compared to the pre-IVM control, while there was no difference compared to the 6 h IVM control. Ten nanomolar and 1 µM BPS significantly decreased *AMH* (by 49% and 63% respectively, *p* = 0.03 and *p* ≤ 0.001 respectively) after 6 h IVM, while the same tendency was observed in the control condition (*p* = 0.0556). BPS treatment had no effect on the other analysed genes.

Phosphorylation of MAPK3/1 was investigated in 10 nM and 1 µM BPS-treated oocytes after 6 h IVM (Supplementary Figure S2). There were no differences in MAPK3/1 phosphorylation between the 0 h control and 6 h IVM control conditions. After 6 h IVM, 1 µM BPS induced a 5-fold increase in phosphorylation compared to the control (*p* = 0.04).

## 3. Discussion

This study aimed to evaluate the effects of BPS, a structural analogue of BPA, on ovine oocyte developmental competence during IVM. For the first time, we reported that low environmental doses of BPS during IVM negatively affected early developmental oocyte competence in ewes.

### 3.1. BPS Reduced Oocyte Nuclear Maturation

In the present study, 10 µM BPS reduced the proportion of mature oocytes after IVM. This result is consistent with several studies that reported the deleterious effects of BPS on oocyte maturation. Indeed, BPS alters metaphase II achievement in porcine oocyte [36] and modifies spindle morphology and chromosome alignment in bovine metaphase II oocytes without affecting the rate of mature oocytes [37]. Comparatively, BPA decreases the rates of oocytes that reach metaphase II in humans [24], mice [48,49,50], rats [51], swine [23,52] and bovines [25]. In our study, only the highest BPS dose decreased the maturation rate. This finding is consistent with studies that reported decreases in oocyte maturation rates usually after exposure to high BPA or BPS concentrations [24,36,37,51,52]. Lower BPA and BPS concentrations do not promote meiotic arrest in rat or bovine oocyte [37,46]. However, BPS does alter spindle morphology and chromosome misalignment in bovine oocytes [37]. BPA causes similar effects in mice [34,53] and increases meiotic aneuploidy in mice [54]. Nevertheless, these discrepancies may be attributed to differences in sensitivity among species. Indeed, in a sensitive in vitro foetal model, human gonadal cells were 10- to 100-fold more sensitive to BPA, BPS and BPF than rodent [1]. Future studies should focus on spindle morphology after BPS exposure, even when there is no observed effect on oocyte maturation. Such potential alterations could help explain the BPS effect on cleavage rates. However, BPS effects on blastocyst rates are normalised to cleaved embryos and thus these changes are not related to potential defects in oocyte maturation.

### 3.2. Low BPS Doses Impaired in vitro Ovine Early Developmental Oocyte Competence

In the present study, we showed that 1 µM BPS decreased the embryo cleavage rate. Moreover, both 10 nM and 1 µM BPS reduced the blastocyst rate. These results are consistent with the literature. Indeed, in vivo BPS exposure in mice (10–100 µg/kg body weight, for 3–4 weeks), reduces the blastocyst rate, increases embryo arrest [35] and reduces the cleavage rate [34]. Exposure of zebrafish to BPS in the water reduces offspring hatchability, even if the hatching occurs in clean water. These data suggest a BPS-mediated effect on gamete quality [29]. Concerning BPA, treatment during IVM in bovines impairs embryo development [55]. A similar phenomenon occurs in *Xenopus laevis* [56]. Porcine parthenotes exposed to 100 µM BPA also exhibit impaired blastocyst formation [57]. Epidemiologic studies in humans also report that high BPA levels may alter oocyte quality and rates of normally fertilised oocytes [17,18,24,58]. Nevertheless, studies in the literature usually report bisphenol effects using high doses or long treatment durations. Our particular interest in the present study was to examine effects after short-term (24 h) BPS treatment during IVM on both cleavage and blastocyst rates, assessed 2 and 7 days after the end of the treatment, respectively. The proportion of > 4-cell embryos and hatched blastocysts were especially reduced at day 2 and 7, respectively. These data suggest that BPS could delay embryo development. Moreover, 10 nM BPS, which corresponds to a low environmental dose [59,60], was sufficient to alter the blastocyst rate in ewes. Our results therefore show that a low dose BPS 10 nM acted solely on blastocyst rates, while a higher dose (BPS 1 µM) acted on both cleavage rate and blastocyst rates. This finding suggests that independent mechanisms of action might be triggered depending on the concentration of BPS used, likely related to the activation of several receptors according to BPS varying affinity with these receptors (GPER, ERRγ, ESR1, ESR2 might be involved, or even other receptors or protein interactions that would need to be identified). Such independent mechanisms of action might also explain why we observed nonlinear effect of BPS. Indeed, the alteration of blastocyst rate reported after 10 nM treatment might be due to a binding with the receptor with the highest affinity with BPS. On the contrary, at the higher BPS 100 nM concentration, the mild activation of several pathways might have antagonistic effect that could explain the absence of phenotype observed. At even higher concentration (BPS 1 µM), the activation of other pathways is likely stronger (a higher concentration being able to compensate for a lower affinity for other receptors) and led to an altered phenotype. Taken together, our results show that BPS exposure altered oocyte quality by several mechanisms altering oocyte maturation rate or cleavage rate and/or blastocyst rates. Because these phenotypes all lead to a reduction in pregnancy rate either by preventing the fertilization or by promoting pregnancy loss, this finding could have implications for human reproduction where caution might be advocated. Compared to the concentrations assessed in this study, in human populations, BPS is reported to average 0.02–21 ng/mL (0.02–84 nM) in urine, values that indicate that it reaches the same concentration range as BPA [28]. Further studies should compare whether the same severity of effect is observed after BPA or BPS treatment in ewe oocyte developmental competence.

### 3.3. BPS Reduced CC Progesterone Secretion

In the present study, BPS significantly reduced CC progesterone secretion. This effect is not consistent with previous studies in granulosa cells. Indeed, granulosa cells exposed to BPS at similar concentrations to those used in this study (up to 10 µM) did not exhibit reduced progesterone secretion in swine [61] or bovines [62]. Concerning BPA effects, a study reported that 100 µM BPA decreased progesterone production by FSH-stimulated porcine COCs after 44 h culture [23] whereas in human cumulus granulosa cells the same concentration of BPA increased progesterone secretion after 48 h culture [63]. There are also consistent reports of decreases in progesterone secretion after porcine granulosa cells are exposed to BPA [20,22,64]. Notably, contrasting effects were reported between low (increased progesterone secretion) and high (decreased progesterone secretion) BPA concentrations in porcine granulosa cells [20]. Moreover, BPA also decreases progesterone secretion in both rat and human granulosa cells [65,66]. The BPS effect on COC progesterone secretion could depend on species cell sensitivity and on bisphenols concentration, but also on hormonal cell treatment. Nevertheless, progesterone secretion is not a critical function in CC compared to granulosa cells, even though they are capable of producing steroids. Thus, the deleterious effects of BPS observed on oocyte developmental competence might not be linked to steroid secretion.

### 3.4. BPS Mechanism of Action

Bisphenols, notably BPA, reportedly act through estrogen receptors. Thus, we assessed the gene expression of steroid receptors in CC, as well as expression of genes involved in oocyte quality. Concerning steroid receptors, *ESR1*, *GPER* and *PR* expression increased during IVM, while *ESR2* expression decreased. *ESR1* was more highly expressed than *ESR2* in CC. In this work, we did not observe any BPS effect on estrogen receptors’, namely *ESR1*, *ESR2* and *GPER*, mRNA expression. These data are consistent with studies in porcine CCs, where BPS does not affect *ESR1* and *ESR2* during IVM. The only effect is a decrease in *ESR2* expression for a higher BPS dose (30 µM) [36]. Of note, the experiments reported here were performed in an oestradiol-containing medium. Due to the oestradiol higher affinity for ESR1 and ESR2, it is likely that BPS effects reported here are unrelated to these receptors. We also reported an effect of BPS on decreased *PR* expression. A relevant decrease in the progesterone receptor-associated gene expression with a concomitant abnormal PGR localisation was reported in mice uterine tissue after BPA exposure [67].

In this paper, phosphorylation of MAPK3/1 was investigated in 10 nM and 1 µM BPS-treated oocytes after 6 h IVM (Supplementary Figure S2). After 6 h IVM, 1 µM BPS induced a 5-fold increase in phosphorylation compared to the control (*p* = 0.04). This result might be relevant with the decreased cleavage rate reported at this concentration in case the increase of MAPK phosphorylation is irreversible and oocytes are not capable to break the second meiotic block. As described in the literature, MAPK3/1 is an abundant protein in vertebrate oocytes, and its activation is required during oocyte maturation [68], explaining why we chose to investigate this pathway. This signalling pathway is implicated in meiotic regulation and spindle organisation [69,70]. Comparatively, BPA was reported to disrupt MAPK3/1 signalling. In metaphase II oocyte from in vitro-grown preantral follicles, 4.5 and 45 µM BPA reduced MAPK3 phosphorylation [51]. However, low doses of BPA (under 1µM) activates rapidly MAPK3/1 signalling in human placental and ovarian cancer cells [71,72]. Nevertheless, as we could not evidence the expected increase in MAPK3/1 phosphorylation between the 0 h control and 6 h IVM control conditions, and caution is needed to interpret these data. Moreover, given the fact that we studied a single endpoint, we could not suggest that BPS has a direct activatory effect on MAPK3/1, but that BPS led to MAPK3/1 activation. Further studies are required to demonstrate that the deleterious BPS effects on ewe oocyte quality could partly occur through the MAPK3/1 signalling pathway disruption. Other signalling pathways need investigating to decipher BPS mechanisms of action.

Notably, the effects reported in this paper were not dose-dependent; they sometimes occurred for one concentration but not necessarily for all higher concentrations. A non-monotonic response to endocrine disruptors was previously reported and is now well established [73,74]. This phenomenon also occurs for BPA in human [75] and mouse cells [76]. The comparison between the effects observed in this study with those reported in other species at similar doses might indicate that ewe COC is a more sensitive model for reproductive toxicant examination because effects are reported at lower doses in ewe compared to bovine or porcine cells [22,37,62]. Finally, there was no observed effect on cell viability at the tested concentrations. These data are consistent with previous results that also report no effect on porcine COC viability [36]. This absence of an effect on cell viability requires additional investigation into the BPS-specific mechanisms of action because the observed effects are not linked to the secondary effect of cell mortality.

Several limitations occurred in this study, related to the use of ovine ovaries collected in slaughterhouse. Indeed, health and metabolic status of ewes are unknown. Moreover, due to the limited number of COCs collected during one experiment (300 COCs from 260 ovaries), it was challenging to evaluate simultaneously BPS and BPA. TCM199 medium used for in vitro maturation also contained phenol red, which has estrogenic properties and might not be ideal to evaluate endocrine-disrupting properties of compounds such as bisphenols. Nevertheless, the IVM medium used also contained oestradiol, which has stronger estrogenic properties and renders negligible the effect of phenol red. Studying BPS effects in an oestradiol-containing medium is necessary if the physiological relevance is the objective, as the oocyte is surrounded by oestradiol especially during late maturation. Here, we described the acute effects (24 h in vitro maturation) of low dose BPS on the final step of terminal oogenesis in adult ewe. A large range of BPS doses were evaluated and a high number of COC were used in the experiments. Sheep as a mono-ovulating species is a relevant model to study oocyte competence. We showed here that BPS reduced COC maturation, cleavage and blastocyst rates, and COC progesterone secretion, and therefore BPS impaired oocyte developmental competence. These data suggest that BPS might not be a safe alternative for BPA, which could have a high relevance for human health and reproduction.

## 4. Materials and Methods

### 4.1. Ethics

No experiments with living animals were performed in this study.

### 4.2. Chemicals and Antibody

Unless otherwise stated in the text, all chemicals, including BPS (4,4′-sulfonyldiphenol), were obtained from Sigma-Aldrich (Saint Quentin Fallavier, France). The utilized primary antibodies are indicated in Additional Table 1. Horseradish peroxidase (HRP)-conjugated anti-rabbit and anti-mouse antibodies were purchased from Perkin Elmer (Courtaboeuf, France).

### 4.3. Biological Material

Ovaries from adult ewes in reproductive age were collected at a local slaughterhouse and transported to the laboratory within 2–3 h in saline solution (9‰ NaCl) with penicillin (1667 IU/L) at a temperature of approximately 35 °C. Ovine immature COCs were retrieved from ovaries by aspiration of healthy antral follicles from 1 to 3 mm in diameter using a 20 gauge needle linked to a vacuum pump and a 50 mL falcon tube. Only good morphological quality COCs, with complete and compact cumulus layers and a homogenous cytoplasm were selected (Supplementary Figure S1A) and washed in HEPES buffered-TCM199 medium (H-TCM199) with 0.04% bovine serum albumin (BSA) and 25 μg/mL gentamycin.

### 4.4. IVM

IVM was performed for 24 h in 4-well polystyrene dishes (Nunclon surface treated, Nunc, ThermoFischer Scientific, Illkirch, France) with groups of 30–60 COCs in 500 μL bicarbonate-buffered TCM199 that contained Earle’s salts and L-glutamine (0.7 mM) supplemented with 10% (*v*/*v*) foetal calf serum (FCS), 10 ng/mL epidermal growth factor (EGF), 100 µM cysteamine, 1 µg/mL 17 β-oestradiol, 10 µg/mL follicle-stimulating hormone (FSH), 12 µg/mL luteinising hormone (LH) (Reprobiol, Lièges, Belgium) and 10 µg/mL gentamycin in the presence or absence of BPS (1 nM, 10 nM, 100 nM, 1 μM or 10 μM) at 38.8 °C in a humidified atmosphere that contained 5% CO_2_. BPS stock solution was dissolved in absolute ethanol (Carlo Erba, Val de Reuil, France). The ethanol dilution in the highest BPS condition (10 µM) is 1/10,000 (0.01%) and is 1/100,000,000 in the lowest BPS condition (1 nM, 0.000001%). As the required ethanol concentration greatly varies between conditions (10,000-fold), we chose not to supplement all conditions with the highest ethanol concentration. Nevertheless, we checked that three ethanol conditions (1/10,000, 1/100,000 and 1/100,000,000, corresponding to BPS 10 µM, 1 µM and 1 nM, respectively) had no effect on developmental rates, neither on cleavage rates nor on blastocyst rates. The absence of BPS 100 nM effects also suggest an absence of effect observed with ethanol 1/1,000,000. After 24 h IVM for COC viability, oocyte meiotic maturation stage, and after 6 h IVM for CC gene expression and oocyte signalling pathway experiments, oocytes were stripped of surrounding CC and cells and spent IVM media were stored at −80 °C until analysis.

### 4.5. IVF and IVD

Thirty to fifty mature COC were fertilised in 500 µL IVF medium (synthetic oviduct fluid, SOF) [77], without BSA and supplemented with gentamycin (40 µg/mL), heparin (15 µg/mL) and 10% inactivated estrous sheep serum) with 3 × 10^6^ spermatozoa/mL. A semen pool from five ram ejaculates diluted in IVF medium with Hepes (10 mM) was used throughout the experiments. Motile spermatozoa were separated by centrifugation (15 min at 700 g) with a 2 mL Percoll (Pharmacia, Uppsala, Sweden) discontinuous density gradient (45%/90%). Spermatozoa and COC were coincubated for 24 h at 38.8 °C in a humidified atmosphere of 5% CO_2_ in air. After CC removal, presumptive zygotes were washed. IVD was performed in a drop of modified SOF (as described above) with 3‰ BSA (*w*/*v*, fraction V, Sigma-Aldrich#A9647) and covered with mineral oil. The cleavage rate and embryo development were assessed at day 2 and 7 post-IVF, respectively. At day 2 of IVD, after cleaved embryo counting using a Zeiss inverted microscope (Zeiss, Germany), 10% FCS was added to the drop of medium. The cleavage rate was defined as the total number of cleaved embryos divided by the total number of fertilised COC. Embryos were analysed 7 days (168 h) after IVF commenced, and the total number of blastocysts (early unexpanded blastocysts, expanded blastocysts and hatched blastocysts) was recorded. All blastocyst rates were defined as the total number of blastocysts divided by the number of cleaved embryos. Experiments were repeated eight times with 30–50 COCs per IVM condition per experiment. The effect of BPS on oocyte quality, and therefore on blastocyst rate, was our main focus, justifying why we assessed all five BPS concentrations (1 nM, 10 nM, 100 nM, 1 μM or 10 μM). We then investigated whether the effects observed on blastocyst rates could be indirect consequences of BPS effects on other parameters, such as oocyte maturation or cell viability. We therefore assessed the effects of the BPS concentrations exhibiting an effect on blastocyst rates and on other parameters (oocyte maturation, cell viability, etc.), explaining why not all five BPS concentrations were used in these experiments.

Embryos were fixed in 4% paraformaldehyde, stained with Hoechst 33342 (1 µg/mL) and observed using a confocal LSM 700 Zeiss microscope and Zen software (2009 Light Edition). The number of cells in each embryo was counted using interactive microscopy image analysis software (Imaris, Bit plane, Switzerland). Degraded embryos without visible chromatin staining were not considered. This experiment was repeated 5 times.

### 4.6. Analysis of Oocyte Nuclear Meiotic Maturation Stages

After 24 h IVM, denuded oocytes were fixed in 4% paraformaldehyde (in 1X phosphate-buffered saline, PBS) and stained with Hoechst 33342 (10 mg/mL) to label oocyte chromatin and determine oocyte meiotic status, as detailed elsewhere [78,79]. Briefly, oocytes at germinal vesicle, germinal vesicle breakdown, metaphase I, and anaphase I stages were considered to be immature. The oocytes that had progressed to telophase I and metaphase II were considered to be mature. Experiments were repeated three times with 30–60 COC per condition per experiment.

### 4.7. Cell Viability Analysis

COC viability was first assessed using spent 24 h IVM media using the Bioluminescence Cytotoxicity Assay Kit (Medical and Biological Laboratories, Woburn, USA) to measure adenylate kinase activity according to the supplier’s instructions, with a luminometer (Tristar² S LB 942, Multimode Reader, Berthold Technologies). Adenylate kinase activity of the spent media correlates to the rate of dead cells. This level was normalised by the COC number in each well. Data are expressed as mean ± standard error of the mean (SEM) of 11 independent cultures as light relative units per COC. To confirm this first assay, CC and oocyte viability was also assessed using the Live/Dead Viability/Cytotoxicity Kit for mammalian cells (Life Technologies, Cergy Pontoise, France) according to the supplier’s instructions after removing surrounding CC. Experiments were repeated three times with 50–60 COC per condition.

### 4.8. Progesterone Assay

After 24 h of maturation in the presence or absence of BPS (1 nM, 10 nM, 100 nM, 1 µM or 10 µM), supernatants were stored at −20 °C until processing. The concentration of progesterone was determined in the culture-spent media using an ELISA protocol, as previously described [80]. The absorbance was measured at 405 nm with the Sunrise-basic plate reader spectrophotometer (TECAN Life Sciences, Switzerland) and the Magellan software. For progesterone concentrations ranging from 0.25 to 32 ng/mL, the intra-assay coefficient of variation (CV) averaged <10%. Progesterone secreted by COCs in each well was normalised by the number of COCs in the well. The results of six independent experiments are expressed in ng of progesterone secreted per COC.

### 4.9. Gene Expression in CC

#### 4.9.1. RNA Extraction and Reverse Transcription

Three independent experiments with 3–4 independent samples containing 30 CC collected after 6 h IVM (therefore, non-expanded CC) in presence or absence of BPS (10 nM or 1 µM) were used for transcriptomic analysis by real-time polymerase chain reaction (qPCR). Briefly, total RNA was extracted from CCs using Tri reagent (T9424, Cergy Pontoise, France) and treated using XS RNA Nucleospin (Macherey Nagel, France), following the manufacturer’s instructions. Subsequently, the RNA concentration was determined using a NanoDrop ND-1000 spectrophotometer (Nyxor Biotech, Paris, France), and RNA integrity was checked by electrophoresis. DNase treatment and reverse transcription were performed on 150 ng total extracted CC RNA using the Maxima First Strand cDNA Synthesis kit (Thermo-Fisher Scientific), according to the manufacturer’s recommendations.

#### 4.9.2. qPCR Amplification

qPCR was performed as previously described [81]. The geometric mean of two housekeeping genes (ribosomal protein L19 (*RPL19*) and beta-actin (*ACTB*)) was used to normalise gene expression. The relative amounts of gene transcripts (R) were calculated according to the equation:

(1) $$R\;=\;\frac{\left(E_{gene}^{-Ct\;gene}\right)}{\left(\mathrm{geometric}\;\mathrm{mean}\;\left(E_{ACTB}^{-Ct\;ACTB};\;E_{RPL19}^{-Ct\;RPL19}\right)\right)\;}$$

where E is the primer efficiency (Table 1) and Ct is the cycle threshold.

Expression of 11 genes (Table 3) was assessed, including steroid and hormonal receptors, described as potential targets of BPA: estrogen receptor 1 (ESR1), estrogen receptor 2 (ESR2), G protein-coupled estrogen receptor 1 (GPER), progesterone receptor (PR), androgen receptor (AR), follicle-stimulating hormone receptor (FSHR) and Anti-Mullerian Hormone Receptor Type 2 (AMHR2). The gene expression of genes involved in oocyte competence, such as the transcription factor Peroxisome proliferator-activated receptor gamma (PPARG), the ovarian reserve marker Anti-Mullerian Hormone (AMH) and bone morphogenetic protein receptors (BMPR1A and BMPR1B), were also evaluated. 

### 4.10. Western Blot Analysis

Proteins were extracted from groups of 30 oocytes, and western blotting was performed as previously described [82]. Antibody dilutions were 1:1000 for phospho-MAPK3/1 and total MAPK3/1 (Supplementary Table S1). After several washes in Tris-buffered saline with Tween 20 (TBST), immunoreactivity was detected using anti-rabbit HRP-conjugated secondary antibodies (diluted 1:10,000). Protein bands were visualised using enhanced chemiluminescence ECL (West Dura; Thermo Scientific, Courtaboeuf, France). A GeneGnome charge-coupled device camera (Syngene, Cambridge, United Kingdom) and Genesys 1.5.4 software (Syngene) were used to acquire the ECL signal. Analysis of signal intensity was performed using GeneTools 4.01 software (Syngene). Three independent experiments were performed, and nine independent samples of oocytes per IVM condition were analysed. Results are expressed in arbitrary units as the ratio of phosphorylated to total protein signal intensity (Supplementary Figure S2).

### 4.11. Statistical Analysis

Statistical analyses, considering both treatment and experiment effects, were performed for all parameters. The distribution of maturation stages, cleavage rates and blastocyst rates were compared by logistic regression analysis using a generalised linear model (R package Rcmdr; [83]) in R version 3.5.3 [84]. Estimated least-square means (lsmeans) for the development rates by the logistic regression model (R package lsmeans; [85]) are provided in Supplementary Table S2. The number of blastocyst cells, COC viability, progesterone concentration and messenger RNA (mRNA) and protein expression levels were compared among the groups using non-parametric one-way analysis of variance (ANOVA), where four conditions were compared (R package lmPerm; [86]), with the Tukey post-hoc test (R package nparcomp; [87]). A difference with *p* ≤ 0.05 was considered significant, and 0.05 ≥ *p* ≤ 0.10 was considered to be a trend.

## 5. Conclusions

In conclusion, we showed that environmental doses of BPS, a structural analogue of BPA, impaired oocyte developmental competence in the ewe. In addition to a reduction in blastocyst rates, we also observed a reduction in the cleavage rate, oocyte maturation rate and progesterone secretion. Due to the presence of oestradiol in the culture medium, those effects could be unrelated to estrogen receptors. Further studies are required to investigate whether BPS affects MAPK3/1 signalling and to determine if such a mechanism could be related to the decrease in oocyte maturation rate.

## Figures and Tables

**Table 1:** Oocyte nuclear maturation stage.

	N Oocyte	Mature Oocyte %
Control	152	88.2% ± 2.6%
BPS 1 nM	117	87.2% ± 3.1%
BPS 1 µM	117	82.8% ± 3.5%
BPS 10 µM	171	76.7% ± 3.2% *

mature oocyte %: % of telophase I and metaphase II oocytes estimated by the logistic regression model; * indicates a significant difference *p* < 0.05.

**Table 2:** Day 6 embryo cell number.

	N Embryo	Cell Number
Control	70	28.3 ± 2.9
BPS 10 nM	75	33.1 ± 3.4
BPS 1 µM	63	22.2 ± 2.3

**Table 3:** Primer sequences used for the qRT-PCR analysis.

Abbreviation	Gene	Forward Primer	Reverse Primer	bp	E
*FSHR*	Follicle stimulating hormone receptor	5′-GGGCCAAGTCAACTTACCACT-3′	5-TGCAAATTGGATGAAGGTCA-3′	144	1.89
*AMHR2*	Anti-Mullerian Hormone Receptor Type 2	5′-GAAAAAGGGCCTTGCTGAA-3′	5′-CAGGACTGCTCACCTTGCT-3′	113	1.83
*PPARG*	Peroxisome proliferator-activated receptor gamma	5′-ATGTCCTCAATGGGCTTCAC-3′	5′-GTGAAGTTCAACGCACTGGA-3′	113	1.87
*ESR1*	Estrogen receptor 1	5′-GGTTCCGTATGATGAATCT-3′	5′-CAAGGTGTCTGTGATCTT-3′	158	1.96
*ESR2*	Estrogen receptor 2	5′-GTCGGTTCTTATCTATGGTA-3′	5′-ACTATGGAGTCTGGTCAT-3′	114	1.99
*PR*	Progesterone receptor	5′-AGTCATCATTCTATTCATTATGC-3′	5′-TGGCTTCTTAGTCCTTCT-3′	142	1.98
*AR*	Androgen receptor	5′-CCTTCACCAATGTCAACT-3′	5′-ATCCACTGGAATAATGCTAA-3′	200	1.90
*GPER*	G protein coupled estrogen receptor 1	5′-TCCCCGACCTGTACTTCATC-3′	5′-GAGGAAGAAGACGCTGCTGT-3′	167	1.98
*BMPR1A*	Bone morphogenetic protein receptor type 1A	5′-TGTCGGACCAACTTATGTAACC-3′	5′-TGAGCAAAGCCAGCCATCG-3′	100	1.92
*BMPR1B*	Bone morphogenetic protein receptor type 1B	5′-TCTTGAGGCAGGATTGTGAGC-3′	5′-GGTGGAGCAGTGACGAGTG-3′	77	1.93
*AMH*	Anti-müllerian hormone	5′-GTGGTGCTGCTGCTAAAGATG-3′	5′-TCGGACAGGCTGATGAGGAG-3′	104	1.88
*ACTB*	Beta Actin	5′-CCAGCACGATGAAGATCAAG-3′	5′-ACATCTGCTGGAAGGTGGAC-3′	102	1.97
*RPL19*	Ribosomal Protein L19	5′-CACAAGCTGAAGGCAGACAA-3′	5′-TGATGATTTCCTCCTTCTTGG-3′	129	1.94

Bp: base pair; E: Efficiency.

## Supplementary Materials

Supplementary materials can be found at https://www.mdpi.com/1422-0067/21/4/1238/s1.
