# Supplementary material for: Bisphenol S Impaired In Vitro Ovine Early Developmental Oocyte Competence
Source: Int J Mol Sci. 2020 Feb 12;21(4):1238. doi: 10.3390/ijms21041238 (PMC7072985; doi:10.3390/ijms21041238)
Supplement: Supplementary file 1 [file ijms-21-01238-s001.pdf]

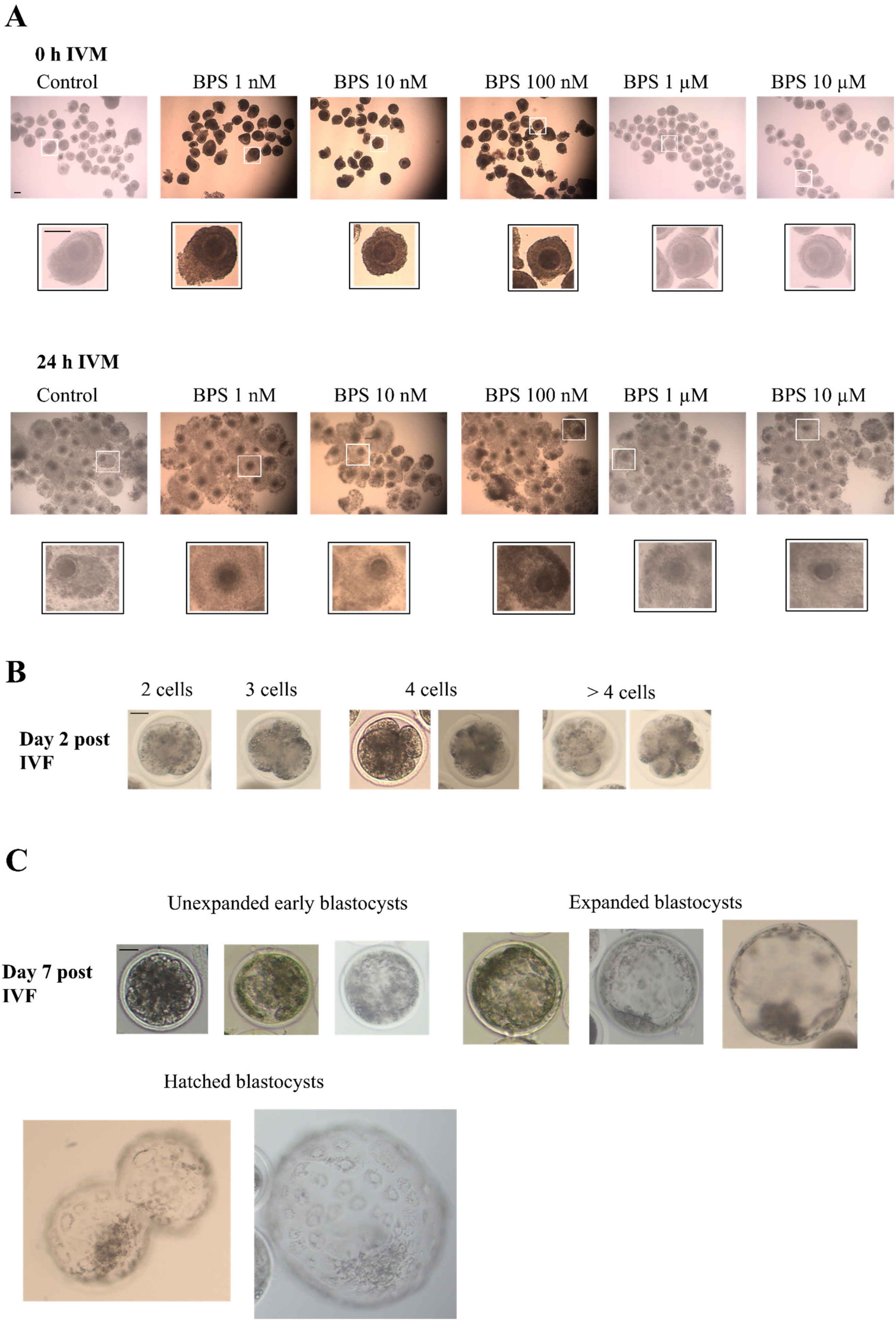
A
0 h IVM
Control
BPS 1 nM
BPS 10 nM
BPS 100 nM
BPS 1 µM
BPS 10 µM
24 h IVM
Control
BPS 1 nM
BPS 10 nM
BPS 100 nM
BPS 1 µM
BPS 10 µM
B
2 cells
3 cells
4 cells
> 4 cells
Day 2 post IVF
C
Unexpanded early blastocysts
Expanded blastocysts
Day 7 post IVF
Hatched blastocysts

Figure S1. Development of cumulus oocyte complexes and embryos during *in vitro* maturation and *in vitro* development post IVF. Morphology was observed under microscopy 40× and 100× for IVM and for IVD respectively. **A**. COC underwent 24 h IVM in the presence or absence of BPS (1 nM, 10 nM, 100 nM, 1 μM or 10 μM). Scale bar represents 100 μm. **B**. Oocytes were subjected to IVF, and the cleavage rate was assessed 2 days after IVF. Embryos with more than 4 cells were distinguished from embryos with 2-4 cells. Scale bar represents 40 μm C. Blastocyst rate was evaluated 7 days after IVF. Blastocysts were classified as unexpanded early, expanded or hatched. Scale bar represents 40 μm.

A

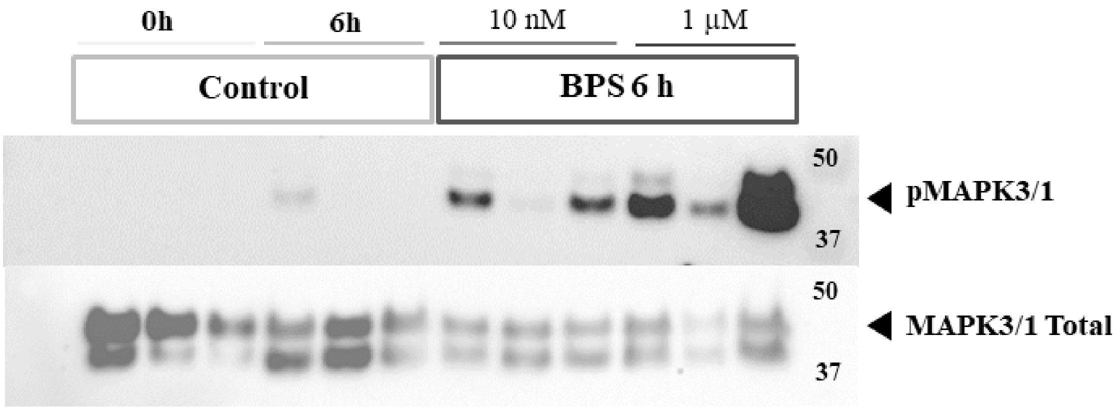

B

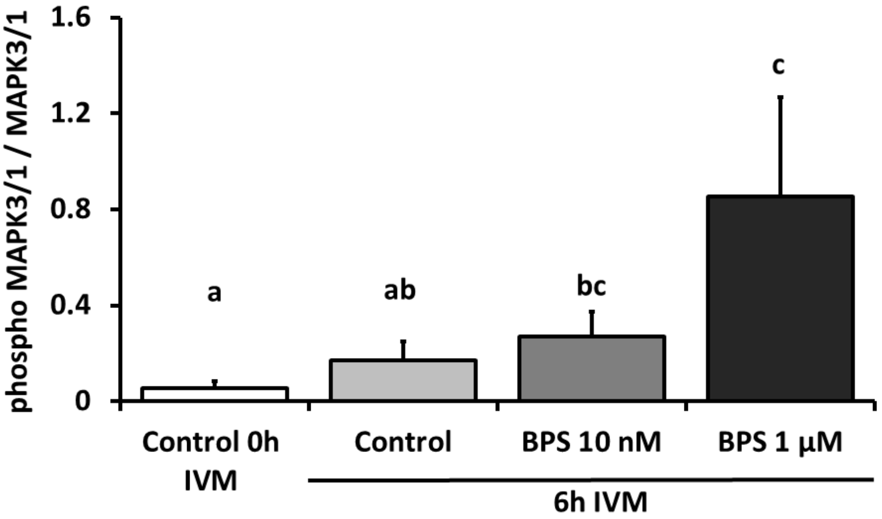

Figure S2. Western blot analysis of MAPK3/1 signalling pathway activation in oocytes after BPS exposure. COC underwent IVM for 6 h in the presence or absence of BPS (10 nM or 1 μM). After 6-h IVM, groups of 30 oocytes were denuded. Proteins were then extracted and separated by electrophoresis on 4–12% (*w*:*v*) sodium dodecyl sulphate polyacrylamide gels. After electrotransfer to nitrocellulose membranes, the proteins were probed with anti-phospho-MAPK3/1 and anti-MAPK3/1 antibodies. **A**. Representative blots and bands are presented in this figure. **B**. Bands on the blots were quantified and the phospho/total protein ratio was calculated. Results are expressed relative to the control as mean ± SEM of nine independent samples per condition. Different letters indicate significant differences ($p \leq 0.05$).

Name	Species Specificity	Source	Supplier (Distributor, Town, Country)	Reference
MAPK3/1 (ERK1/2)	Rat	Rabbit polyclonal antibody	Cell Signaling Technology (Ozyme, Saint Quentin Yvelines, France)	9102
Phospho-MAPK3/1 (ERK1/2) (Thr202/Tyr204, D13.14.4E, XPTM)	Human	Rabbit monoclonal antibody	Cell Signaling Technology (Ozyme, Saint Quentin Yvelines, France)	4370
Vinculine	Human	Mouse monoclonal antibody	Sigma Aldrich Saint Quentin Fallavier	V9131

Table S1: Characteristics of primary antibodies used for western blotting

Table S2. Results of logistic regression analysis and presenting therefore estimated % of the model comparing all conditions at the same time on the effect of BPS during IVM on *in vitro* embryo development after *in vitro* fertilization, and taking into account both treatment and experiment effects (logistic regression model estimated % ± sem)

**Conditions**		**Mature COC**	**Cleaved embryos**	**2-4 cells embryos**	**> 4 cells embryos**	**Blastocysts**	**Unexpanded early blastocysts**	**Expanded Blastocysts**	**Hatched blastocysts**
**Control**	**N**	370	202	81	121	44	10	15	19
	**%**		57.4 ± 6.1	21.6 ± 3.1	33.6 ± 4.7	22.2 ± 3.5	3.8 ± 1.9	7.4 ± 1.9	9.4 ± 2.1
**BPS 1nM**	**N**	281	197	69	128	37	5	16	16
	**%**		71.7 ± 5.4	23.5 ± 3.5	45.6 ± 5.3	18.7 ± 3.2	1.8 ± 1.1	8.0 ± 2.0	8.1 ± 2.0
	***p*-value**		**0.0003**	0.564	**0.0025**	0.3918	0.176	0.819	0.6474
**BPS 10nM**	**N**	298	183	79	104	26	4	12	10
	**%**		61.2 ± 6.1	25.6 ± 3.6	33.3 ± 4.8	14.2 ± 2.9	1.9 ± 1.2	6.4 ± 1.9	5.4 ± 1.7
	***p*-value**		0.335	0.227	0.942	**0.0458**	0.246	0.720	0.150
**BPS 100nM**	**N**	296	167	62	105	33	7	11	15
	**%**		55.0 ± 6.3	19.6 ± 3.1	33.6 ± 4.8	20.4 ± 3.6	4.3 ± 2.3	6.5 ± 2.0	8.9 ± 2.3
	***p*-value**		0.561	0.537	0.980	0.674	0.786	0.752	0.882
**BPS 1uM**	**N**	319	152	77	75	19	6	8	5
	**%**		45.9 ± 6.3	23.0 ± 3.3	21.8 ± 3.8	12.1 ± 2.9	3.3 ± 1.9	5.1 ± 1.8	3.3 ± 1.5
	***p*-value**		**0.0041**	0.657	**0.0008**	**0.0168**	0.814	0.382	**0.0315**
**BPS 10uM**	**N**	321	163	74	89	24	7	12	5
	**%**		50.7 ± 6.3	22.2 ± 3.3	26.7 ± 4.3	14.7 ± 3.1	3.6 ± 1.9	7.2 ± 2.1	3.1 ± 1.4
	**p-value**		***0.0923***	0.842	***0.0574***	***0.0715***	0.918	0.953	**0.0212**

N: Number of samples ; % is the % estimated by the logistic regression model ± sem ; p-value is the logistic regression p-value of the comparison to the control condition ; bold characters indicate significant differences $p \leq 0.05$; italic and bold characters indicate tendencies $0.05 < p < 0.1$
